# Location, location, location: the approach of healthcare professionals in defining the artificially gestated entity

**DOI:** 10.1093/medlaw/fwaf035

**Published:** 2025-09-22

**Authors:** Victoria Adkins

**Affiliations:** School of Law and Criminology, University of Greenwich, London, SE10 9LS, United Kingdom

**Keywords:** artificial placenta, consensus, healthcare professionals, legal status, partial ectogestation, regulation

## Abstract

Clinical trials of artificial placentas are anticipated; however, debate continues over how to define an artificially gestated entity, and little empirical research has explored stakeholder perspectives on this issue. This article presents findings from the first study in England to engage with healthcare professionals’ perspectives. Healthcare professionals, as intermediaries between developers and patients, and clinical experts, are central to shaping technology integration into clinical practice. The analysis presented in this paper frames their views on the artificially gestated entity by different forms of ‘location’. This illustrates how they align the entity with either a newborn or a foetus or propose interim definitions. The lack of consensus amongst healthcare professionals is shown to derive from their reliance on existing legal and medical frameworks. The significance of this article therefore lies in the evidence it provides that the current legal framework in England does not adequately support a consistent definition of an artificially gestated entity. Further, engagement with this stakeholder group reveals the practical implications that ambiguous definitions could have for clinical settings. This article argues that stakeholder groups must collaborate to develop regulatory frameworks for artificial placenta technology that support clinical integration and account for the interplay between law and medical practice.

## I. INTRODUCTION

Partial ectogestation is the process by which an entity is partially gestated outside of the body. Whilst infants born prematurely are currently placed into neonatal intensive care, the invasive nature of this care can often contribute to significant life-long morbidities.^1^ In seeking to improve the outcomes following premature birth, researchers are developing devices which mimic the human placenta.^2^ Whilst some artificial placenta prototypes infuse the entity’s lungs with fluids directly,^3^ the device closest to human clinical trials is known as the ‘Biobag’.^4^ This artificial placenta device, developed by a team at the Children’s Hospital of Philadelphia (CHOP),^5^ involves submerging the entity into artificial amniotic fluid and connecting an oxygenator pump to the blood vessels in the umbilical cord. The purpose of the device is to allow the entity to develop and breathe as it would in a human placenta, thereby eliminating the use of the lungs required in neonatal care. Whilst the Food and Drug Administration Paediatric Advisory Committee in the USA have not yet approved clinical human trials for the CHOP team,^6^ the imminence of such trials remains on the horizon.^7^

The definition of an artificially gestated entity,^8^ the entity that will come to be gestated in an artificial placenta through the process of partial ectogestation, has been the subject of much legal and ethical discourse. Claims have been made that the entity will simply be a neonate,^9^ with artificial placentas depicted as improved methods of neonatal care.^10^ In contrast, questions have been raised as to whether the entity needs a new definition to account for its limited physical association with the world and its physiological characteristics.^11^ Researchers at CHOP have used lambs at the equivalent gestational age to human foetuses of 22–24 weeks, seeking to improve outcomes for premature infants who may or may not receive neonatal intensive care based on their individual prognosis.^12^ However, despite developmental distinctions, the description of these entities as ‘neonates kept in a fetal physiological state’^13^ blurs the boundaries between foetuses and neonates and prompts the question of how they should be defined.

Reliance on existing English law to define an artificially gestated entity is challenged by debates over how artificial placentas might affect abortion rights.^14^ For example, some claim that the technology marks the end of the abortion debate, as the foetus could be transferred to an artificial placenta,^15^ suggesting that a legal right to a termination may no longer be necessary. However, others argue that such surgery is not equivalent to an abortion procedure and undermines the intention behind terminations of pregnancy to not become a parent.^16^ The opposing views in such debates rarely provide clear guidance for legal solutions. In law, a line is often drawn in some middle ground of contentious territory.^17^ Therefore, whilst conversations amongst academics continue to flourish in relation to how a foetus or artificially gestated entity ought to be treated, there is a scarcity of consensus as to how to legally define the artificially gestated entity in English law. In addition, there is minimal consideration of the relationship between legal definitions regarding artificial placentas and the logistics of applying those definitions in medical practice. In seeking to fill this lacuna, this article draws from an empirical study undertaken with healthcare professionals in England, which sought to understand their views on the use and application of artificial placentas.

Healthcare professionals are considered key stakeholders of artificial placenta technology as they will likely oversee the technology’s introduction to patients and serve as a bridge between developers and the public. With the medical profession shown to hold substantial authority within society^18^ and healthcare professionals often considered to hold moral agency,^19^ their perspectives may influence the views of the public and future patients. How healthcare professionals frame the technology could therefore have implications for whether the technology is supported and taken up by patients. If there is a lack of support for the technology, this may hinder its development altogether. Stakeholder engagement further allows for issues to be ‘thrashed out’ prior to their implementation,^20^ providing stakeholders with opportunities to ask questions and giving developers insight into their needs, again increasing the likelihood of support. The experience and knowledge of healthcare professionals place them in an advantageous position to consider how artificial placentas may fit amongst existing medical care. They can shed light on the practical implementation of the technology that developers need to take into account to ensure the technology can be incorporated into reproductive health care.

Some engagement with stakeholders has already been undertaken. For example, empirical studies have been undertaken in the UK with reproductive rights activists^21^, and a study is currently underway seeking the views of parents with experience of neonatal intensive care.^22^ Engagement with healthcare professionals has been undertaken in Australia, although predominantly focusing on viability and the impact on abortion policy.^23^ A study in the Netherlands exploring both healthcare professionals’ and parents’ views in relation to preparation for clinical trials for artificial placentas has recently been published^24^ and shares some synergies with this study, discussed later. The study reported in this article, however, remains the first to solely consider the views of healthcare professionals in England.

Specifically, this article focuses on a theme named ‘Location, location, location’, which illustrates the way in which healthcare professionals in England come to define an artificially gestated entity. Healthcare professionals in this study will be shown to use different forms of ‘location’ to reach their definitions, each of which is informed by current legal and medical frameworks. As will be shown, this results in a lack of consensus amongst the participants as to how to define the artificially gestated entity, mirroring the lack of consensus in academic discourse. An absence of agreement amongst healthcare professionals who will be working closely with pregnant people and the entities subject to artificial gestation does not bode well for a harmonious provision of healthcare. However, the issue lies in the legal and medical frameworks, which cause this lack of consensus, as healthcare professionals rely upon different parts of these frameworks in deciding how to define the artificially gestated entity. The significance of this article therefore lies in the evidence it provides that the current legal framework in England is inadequate for guiding a consistent definition of an artificially gestated entity. Ironically, while the medical profession is sometimes relied upon to fill discretionary gaps in the law,^25^ this expectation may be misplaced in the context of artificial placenta technology. If those who are looked to for regulatory guidance cannot reach consensus due to flaws in the legal framework, then effective regulation may prove elusive.

Beyond exposing the inadequacy of existing frameworks, the analysis also sheds light on how any new definition or understanding of birth, resulting from how the artificially gestated entity is defined, may translate to medical practice. This discussion is largely absent in current theoretical debates, and little attention is given to the critical interaction between legal and medical frameworks, which need to align to protect patients’ rights (ie, doctors must know who is considered a patient in order to know they have a right to treatment). By addressing this gap, this article underscores the clarity required in both legal and medical frameworks if agreement as to the regulatory parameters of this new technology is to be settled.

In what follows, Section II will outline the methods adopted in the study, namely semi-structured interviews and reflexive thematic analysis. Section III will present the analysis of the interview data for the theme ‘Location, location, location’ as detailed above. The section will explore the participants’ use of bodily- and environmental locations, the suggestion of new and interim definitions and the practical implications of how the entity is defined. Section IV then provides a discussion summarizing the analysis and its relevance to existing literature and argues for the development of new frameworks to respond appropriately to artificial placenta technology.

## II. METHODS

The theme presented in this article originates from a qualitative study exploring the views of healthcare professionals in England regarding artificial placentas. An online survey was distributed to healthcare professionals working in midwifery, obstetrics, gynaecology, and reproductive and foetal medicine through 41 NHS Trusts in England. The survey was used to gain preliminary perspectives and to encourage recruitment for semi-structured interviews. Following the closure of the survey, 22 healthcare professionals participated in an interview between December 2020 and March 2021, and it is the interview data that form the basis of the study’s analysis. Interview participants included fourteen midwives, three consultant neonatologists, two consultant obstetricians and gynaecologists, one consultant obstetrician, one antenatal and newborn screening co-ordinator, and one clinical nurse specialist. Participant names have all been replaced with pseudonyms, with some participants selecting their own. For those who did not provide their own pseudonym, a gender-neutral name has been selected.

The semi-structured interviews predominantly took place on the telephone, with one interview taking place on Microsoft Teams (with audio only from the researcher for parity with the telephone interviews). The interviews were framed around a broad interview guide, which was shared with participants and included a citation to the most relevant scientific paper at the time in relation to artificial placentas. This article related to the sealed version of an artificial placenta (the ‘Biobag’) developed by the CHOP team, and a quote from the paper outlining that the current aim of the technology is to improve outcomes ‘for those infants who are already being routinely resuscitated and cared for in neonatal intensive care units’^26^ was included in the interview guide. If pressed for further clarification, details from the paper cited were repeated, such as the use of an oxygenator pump and studies being carried out on lambs at the human gestational equivalence of 22–24 weeks.

Semi-structured interviews were considered appropriate for this study as they allow for the production of rich and descriptive data.^27^ Additionally, the semi-structured nature allowed participants to include additional thoughts and insights beyond the questions asked, whilst also ensuring they remained focused on the research topic. Unfortunately, due to the coronavirus pandemic, the interviews could not take place face-to-face, but this is not considered to have significantly hindered the study.

Reflexive thematic analysis^28^ was employed to analyse the interview data and is a method for ‘identifying, analysing and reporting patterns within data’.^29^ Different epistemological assumptions underpin different iterations of thematic analysis^30^ with reflexive thematic analysis, concerned with researcher subjectivity.^31^ This acknowledges the impact the researcher has on the analysis of the data,^32^ recognizing that the way in which the data is interpreted is specific to the individual undertaking the interpretation. In this study, for example, the researcher approached the data without any specific theory in mind, opting not to use a codebook or coding frame as is often utilized in content analysis.^33^

Reflexive thematic analysis encompasses recursive stages including familiarization, coding, theme development, and writing up. In this study, familiarization included listening to audio recordings of the interviews whilst checking transcripts. Coding, which involves the tagging of specific sections of data that are considered meaningful, was undertaken using NVivo 12. An inductive coding approach was employed,^34^ with many of the codes being semantic in nature to reflect the researcher’s data-driven approach. The codes therefore echoed the content of the data extracted as opposed to latent coding, which is more interpretive.^35^ By examining the relationship between the codes and mapping patterns across them, initial themes were developed. Braun and Clarke explicitly reject the notion that themes ‘emerge’ from data^36^; rather, active choices are made by the researcher as to patterns within the data based on their knowledge of the topic and what they consider relevant to the study. Initial themes were then reviewed against the data set, which reshaped the parameters of the themes. Full theme development unfolded once writing up began as the themes became ‘rich, contextualised stories’.^37^ The final analysis resulted in three themes, one of which, ‘Location, location, location’, is presented in this article. The study received ethical approval from the Health Research Authority (IRAS: 270971) and Royal Holloway’s Ethics Committee (Project ID: 2086).

## III. Analysis: location, location, location

As part of the interviews, participants were asked how they would define the status of an entity in an artificial placenta,^38^ and, if prompted for more clarification, they would be asked whether they would consider the entity a foetus, a newborn, or something different from either. Beyond simply categorizing the status or definitions the participants applied to an artificially gestated entity, the theme centres on the factors that participants took into consideration when making their determinations- the different types of ‘location’ at play. On the one hand, participants focused on ‘bodily-location’, focusing on whether the entity was in or outside of the pregnant person’s body. Alternatively, some participants centred their deliberations more closely on the ‘environmental-location’ of the entity, namely the specific in-utero-like environment in which it would find itself. In relation to the bodily-location approach, some participants considered the exit from the pregnant person’s body (assuming it is born alive) to be enough for the entity to be considered born and therefore aligned with the same status as a newborn. The more inward focus on the environmental-location approach alternatively led some participants to construct the entity as being more akin to a foetus. In addition, a third form of location arises from the analysis, referred to as the ‘contextual-location’. This reflects the way in which the participants were continually questioning where the entity would fit in relation to existing legal and medical models, regardless of reliance on either the bodily or environmental location. Throughout their considerations, participants continuously tried to fit the operation of artificial placentas into current legal and medical contexts and the artificially gestated entity into an already formed construction of what it means to either be born or be in an in-utero-like environment.

In attempting to coincide the bodily and environmental locations, participants in the study also considered the possibility of a new or ‘interim’ definition for the artificially gestated entity. In doing so, it is revealed how the existing legal framework may no longer be reliable. By producing the possibility of an entity gestating in an in-utero-like environment outside of a human body, an artificial placenta challenges legal bright lines that rely upon bodily location. However, as will be shown, reliance on bodily location still remains relevant as participants used this to defend providing some protections to the artificially gestated entity whilst in the artificial placenta.

### A. Bodily location

When considering how they may define an artificially gestated entity, some participants deferred to ‘logic’ that flows from the entity having left the pregnant person’s body. Jay, a midwife, for example, stated:

I suppose, logically, it would have to be a newborn because I guess it has been born from the mum, so it has to be registered as a birth. (Jay, Midwife)

Jay’s adoption of a ‘logic’ to reach their conclusion appeared to be informed by legal processes. Specifically, Jay referred to the registration of birth, which is required after 42 days of a child being born,^39^ and Jay’s reference to the entity being ‘born from the mum’ appears to align with legal definitions of birth. For example, birth is defined under section 4(2)(a) of the Congenital Disabilities (Civil Liability) Act 1976 as the moment in which a child “first has a life separate from his mother”. The Infant Life Preservation Act 1929 further refers to ‘existence independent of its mother’,^40^ and the Births and Deaths Registration Act 1953 defines a stillborn child as ‘a child which has issued forth from its mother…’.^41^ The logic to which Jay referred appears to therefore pertain to the legal significance that is attached to the exit of a foetus from the pregnant person’s body and the legal processes that follow. Case law additionally supports the notion that separation from the pregnant person’s body is significant for the legal treatment of those who have been born, both in civil^42^ and criminal law.^43^ A similar reliance on pre-existing knowledge was also evident from Sacha’s response:

…Obviously when it’s part of its mum then obviously it’s part of her body, but obviously separated from mum it becomes a being in its own right. So really, what I think of it, whether I think of it as it as a gestating foetus or a newborn makes no difference, it’s separated from its mum so therefore its legal status changes. (Sacha, Midwife)

Sacha’s use of the word ‘obviously’ further evidences a reliance on an already established understanding of birth. Interestingly, and unlike Jay, Sacha draws a line between what they may consider the entity to be and the legal implications of the entity’s separation from the pregnant person. Although Sacha never fully outlines their own view, it is the legal implications that take precedence, as can be seen with Sacha’s statement that what they think of the entity ‘makes no difference’. This deference suggests that the matter is to be legally rather than medically determined, indicating that the legal context was informing both Jay and Sacha’s responses.

Reliance on the existing legal framework was particularly evident in Harley’s response below:

I think that I would probably give it the status of a neonate which is difficult because of the laws that we have in—well, I think you’d have to do that because of the laws that we’ve got in England, I'm going to rephrase that…. So, I don’t think you could extrapolate, I think it would be very difficult to extrapolate that once a baby is not part of the woman, you know, they are not a combined entity, in order for it to be comfortable with the law that we currently have in the UK, you would have to define it as a neonate, that it is now part of society because it is no longer part of mum. Therefore, it would have its own rights. (Harley, Consultant Neonatologist)

Whilst responses from other participants suggest that the understanding of birth is taken for granted, Harley appeared to grapple with how they would define an artificially gestated entity and the existing legal framework. In the end, it seems Harley conceded to the existing legal framework for the status of the entity to sit ‘comfortably’ with current understandings of birth.

It should be noted that English law also demands that an entity must show signs of life at birth to be considered born alive. The requirements of the born-alive rule can be understood through legal definitions of when a birth has not occurred. For example, a foetus that is expelled from the pregnant person’s body without showing any signs of life and is 24 weeks of gestation or more is considered a stillborn.^44^ Whilst the birth is legally acknowledged by the requirement for its registration,^45^ the stillborn child is not considered to have legal rights.^46^ Any death of the foetus prior to 24 weeks of gestation is considered a miscarriage, with no requirement of registration.^47^ Therefore, to obtain full legal rights, the child must be born both in terms of its expulsion from the pregnant person’s body and by showing signs of life. Consideration as to whether an artificially gestated entity would be considered born alive was not a prominent discussion point in the interviews, most likely because the discussions were framed around the assumption that the entity does not die during or shortly after childbirth.^48^ Participants who relied on the bodily-location approach may have simply assumed that the entity under discussion was born alive or may have overlooked its relevance in their response to the interview questions. Points at which this assumption was not as clear are discussed later in the analysis.

Beyond reliance on the legal framework, there was also evidence of how the medical context was informing dependency on bodily location. In responding to whether they would consider the entity inside an artificial placenta to have been born, Freda stated:

I think, mmm, I would think yes because I would see the artificial womb as being a more advanced incubator. And it is separated from the mother. You don’t have that umbilical, placental connection, and it has to come out of the womb in some way, whether it’s a caesarean section or vagina delivery, so it has exited the womb, so it has been born. So yes. (Freda, Midwife)

With Freda’s description, the bodily location change signifies physical separation from the pregnant person’s body through a disconnection with the placenta and umbilical cord. Such a description suggests that the bodily location change is not simply a case of being in or out of the pregnant person’s body. Instead, the change signifies that a particular physical link between the entity and the pregnant person has been severed. Warren, in academic literature, has similarly described the end of the physical connection between the pregnant person and the foetus as the end of the ‘unique biological unity’.^49^ The relevance of the bodily location for Freda here is therefore informed by medical descriptions as well as legal ones.^50^ The context of existing medical practice is also apparent in Freda’s statement, as they referred to the technology as an ‘advanced incubator’. An association is therefore made between the artificial placenta and existing neonatal treatment as part of the thought process in defining an artificially gestated entity.

Similarly, a focus on the separation from the pregnant person’s body also allowed participants to frame how the technology itself is viewed in correlation with other treatments:

To me, coming out of the mother is when you’re born. That would be… Yes, that’s quite- yes, I don’t have to think much about that one. And in that case, I feel that the treatment that ectogenesis is more like a very smart incubator. (sic) (Frances, Antenatal and Newborn Screening Co-ordinator)

From Frances’ response, in starting with the existing position that the entity has been born due to its separation from the mother, they were then able to frame artificial placentas (or the process of partial ectogestation, which it undertakes) as akin to another form of medical treatment that treats similarly defined entities, such as newborns. The context of existing medical practice therefore supports the participants’ reliance on the bodily-location approach. In not having to ‘think much about’ whether leaving the pregnant person’s body equates to birth, it is again clear that there was a somewhat taken-for-granted position that separation between the entity and the pregnant person is a settled signifier of birth. Having satisfied themselves of this position, Frances was then able to place an artificial placenta within the same category as an incubator, which also assists with born entities.

The mapping of artificial placentas to existing medical practice is further succinctly captured in the dialogue below:

Researcher: And you would consider it [the artificially gestated entity] to have been born?

Rory: Yeah. All you’re doing is modifying the treatment that you’re giving the baby.

Researcher: And do you think when it comes out of the artificial womb, should that, would you give that any sort of significant label?

Rory: Not really, just like I wouldn’t change the way I talk about a baby when I take it off the ventilator. (Rory, Consultant Neonatologist)

With the response given by Rory, the idea that an artificial placenta is a somewhat different and provocative technology^51^ is diluted, as Rory simply considered it an alternative treatment for a baby. Such a construction is also shared by some academics who consider current neonatal intensive care to be proof that partial ectogestation already exists.^52^ Much like Frances above, in relating artificial placentas to already existing medical devices, Rory could consider an artificially gestated entity in the same way they would consider a ‘baby’ subject to existing forms of neonatal intensive care. This correlates with some developers of artificial placenta technology, who argue that it is simply another means of providing gas exchange, and as such, there is nothing unique about the entity making use of the technology.^53^

Sacha, who, as seen earlier, was conceding to the legal framework, also indicated how the bodily location of the entity and existing medical practice are used in combination to assert the status of the artificially gestated entity:

…I think, yeah I would consider it born if it’s separated from its mum whether that be by caesarean section or normal delivery and then it’s then somewhere else undergoing treatment, whether that be just standard intubation or whether it be in an artificial womb, it has been born and it’s now in a clinically treated environment. (Sacha, Midwife)

Through Sacha’s description, the birth of the entity that is to be subject to artificial gestation is no different than the birth that occurs when other treatments are utilized. Unlike Rory, Sacha explicitly references the entity leaving the pregnant person’s body; however, they nevertheless take a similar approach in considering an artificial placenta to be just one of a variety of possible treatment devices. Again, even when relying on existing medical practice to confirm a status for the artificially gestated entity, the bodily location of the entity (in terms of being outside of the pregnant person’s body) is embedded within these constructions. The existing treatments referred to, such as standard intubation, also abide by and rely upon the significance of the ‘baby’ leaving the pregnant person’s body. Aligning artificial placentas with these existing treatments, and thereby this medical and legal context, results in the status of the entity being determined by its location outside of the pregnant person’s body.

The bodily location of the entity is therefore crucial, for some participants, to the understanding of whether it is considered born. The way in which the significance of bodily location is assumed, without much question, suggests that such an approach is informed by both the legal and medical contexts within which the entity will exist.

### B. Environmental-location

Whilst the bodily-location approach taken by some participants appears to be well supported by current legal and medical understandings of birth, an alternative approach of other participants brought the stability of these frameworks into question. A more inward focus on the immediate surroundings in which the artificially gestated entity would find itself led some participants’ definitions to be guided by the in-utero-like nature of the artificial placenta. For example, when asked whether an artificially gestated entity would be treated like a newborn, Devin stated:

…it depends on the transferred baby, the kind of situation it’s in I guess because a lot of the stuff that makes a newborn a newborn is how the baby is dealing with breathing in air and things. But if it’s still in an in-utero situation it will be still in amniotic fluid of some kind, and depending on the placenta. So, I suppose the care for it would be as if it were an in-utero baby, if that makes sense. (sic) (Devin, Midwife)

From Devin’s response, comparisons between an artificially gestated entity and a newborn are more than just a matter of having left the pregnant person’s body. Devin’s description highlighted that the surroundings into which an artificially gestated entity and a newborn enter after that event are not the same. Notably, Devin’s reference to the in-utero surroundings was linked to the care it would receive as an ‘in-utero baby’ and for this reason, it may not be considered as receiving the same treatment offered to newborns. This contrasts with those relying on the bodily location above who quite firmly equated the treatment given to an entity within an artificial placenta with that given to a premature newborn.

This link between the in-utero-like environment and the treatment of the entity was also of significance to Tate:

Mm, I think, my first reaction was foetus still, because it’s still going into a womb-like sphere and we are still considering it…we’re using the sphere in a way that it would be used inside the person’s own body. So yes I think still a foetus… (Tate, Midwife)

Tate’s reference to the entity being in a ‘womb-like sphere’ stands in stark contrast to Freda’s earlier description of how the entity has ‘exited the womb’ to emphasize its bodily-location change. Unlike those who focus on the similarity between a newborn and an artificially gestated entity due to both having left the pregnant person’s body, Tate’s expression of the technology being used in the same way as a womb meant the entity was considered more akin to a foetus. How the technology is constructed, whether as an ‘advanced incubator’ or another ‘in-utero environment’, therefore influenced whether the entity is viewed as a foetus or newborn.

Further, biological developments played a role in Lucy’s conclusion that the artificially gestated entity would not be considered born:

Researcher: So when would you consider it to have been born?

Lucy: Because it has to, there’s so many complicated processes that happen in foetal development that until it reached term I guess I wouldn’t, ah god I don’t know because what if it was born prematurely? I think so long as there would be a like a reason, a biological reason to keep that baby in utero, if it could be in utero, even if it was in artificial utero, really there would be no, no foetal development carries on until a baby is 40 weeks. There are differences between babies born at 37 weeks in terms of development and 40 weeks, so really they should be kept in there until 40 weeks and not considered to be born, until they’re taken out. That’s what I would probably say. (Lucy, Midwife)

Lucy’s consideration of biological developments, along with the entity being in an ‘in-utero situation’ or in ‘artificial utero’, draws a contrast with a newborn who is entirely ex utero and must breathe through their lungs.^54^ As stated above, English law does require that a foetus exhibit signs of life in addition to leaving the pregnant person’s body; therefore, developmental factors play a role when assigning legal personhood. How signs of life are exhibited has also been a catalyst for debate amongst scholars regarding the status of an artificially gestated entity. Alghrani and Brazier have suggested that if the aid of a ventilator can be expanded to include the assistance that an artificial placenta would offer, then the artificially gestated entity could be considered born alive in the same way as other premature foetuses.^55^ Following the definition of live birth provided by the World Health Organization,^56^ Colgrove also argues that the entity is simply a different kind of newborn and exhibits signs of life through its beating heart.^57^ This is also supported by researchers who outline that the foetal heart must have the capacity and tolerance for the artificial placenta system in order for the technology to be successful.^58^ Romanis, on the other hand, distinguishes the entity from a foetus and newborn altogether on the basis that certain biological adaptations (such as using their lungs to breathe) have not occurred, which in their view means that live birth has not been satisfied.^59^

Lucy’s approach appeared to echo that of Romanis in playing closer attention to the physiology of the entity. However, whilst Lucy suggested that remaining in an in-utero-like environment, albeit an artificial one, would, in their view, mean the entity has not been born, they acknowledged how this went against existing conceptualizations of birth with reference to premature babies. Currently, premature babies are considered born when they leave the pregnant person’s body, providing they exhibit signs of life. How well they do this, alongside other factors such as their gestational age, determines whether they receive neonatal intensive care.^60^ Without explicitly referencing whether the entity is considered to be alive, in distinguishing between the entity and premature babies, Lucy appeared to focus on the entity still undergoing in-utero development, which a premature baby (not artificially gestated) cannot do. Kingma and Finn, building upon Romanis’ claim that the entity is neither a foetus nor a newborn, have similarly drawn out the distinction that Lucy identifies between premature babies (newborns) and artificially gestated entities.^61^ They argue that the artificially gestated entity is not “physiologically” born because it does not breathe, maintain single-pump heart function^62^ and retains the umbilical cord, amnion, and chorion.^63^ As such, the only real similarity it shares with a newborn is its bodily location, having exited the pregnant person’s body.^64^

Devin, a midwife, also discussed the in-utero-like environment of the artificial placenta and attempted to map this environmental location to existing legal frameworks:

But if this artificial womb is trying to replicate a human womb, then surely everything that a human womb has should just be copied over to this artificial womb? And I suppose if, technically, the baby hasn’t been born yet, it’s just been transferred then it technically still isn’t born, it’s still in utero even though it’s an artificial one, then maybe the laws just kind of copy over. (sic) (Devin, Midwife)

Devin’s consideration of the artificial placenta being a mere replication of the human womb led them to determine that the legal framework relating to a human womb would simply be translated to the new device. There are several pieces of legislation that could be connected to the womb, particularly during pregnancy, including those that regulate when a pregnancy can be terminated, such as the Infant Life Preservation Act 1929, the Abortion Act 1967, and the Offences Against the Person Act 1861. Even when participants are not likening the artificially gestated entity to a newborn, as with those focusing on bodily location, participants were still attempting to fit the entity into the pre-existing legal framework. However, the part of the framework being deferred to when considering the environmental location of the entity is that which pertains to foetuses. This would have a significantly different outcome, in terms of how the entity may be treated, than the legal framework relating to newborns, as the artificially gestated entity would not be given the rights assigned to legally recognized persons.^65^ Additionally, it is not necessarily plausible that the legal framework governing foetuses could be translated to the artificially gestated entity. The Infant Life (Preservation) Act 1929, for example, refers to causing a child to die ‘before it has an existence independent of its mother’^66^ and in being in the artificial placenta, the entity would arguably already exist independently of its mother and not benefit from this legal protection.^67^

The examination of the development of the artificially gestated entity from a medical perspective, under the environmental location, leads to its legal recognition as a foetus. Conversely, pitching the artificial placenta as akin to other medical devices like incubators, from the bodily-location perspective, leads to the entity being legally recognized as a newborn. Therefore, despite reliance on the same medical and legal contexts, the bodily-location and environmental-location lead to opposing definitions of the entity, and selective parts of those contexts are relied upon to reach different conclusions. The varied approaches adopted by the participants not only illustrate a lack of consensus amongst healthcare professionals but also expose the fragility of the current legislative and medical frameworks. The prospect of artificial placentas creates a circumstance where it will no longer be the case that being within an in-utero-like environment will always be synonymous with being inside the pregnant persons’ body, and as a result, entities outside of a human body may be physiologically different and continue their development in different ways.

### C. New and interim definitions

Participants who focused on bodily location were quite easily able to designate a status to the artificially gestated entity in considering it no different from a newborn. Those that focused on the environmental location made closer connections between the artificially gestated entity and a foetus. However, the level of certainty with this account was not as strong. As with Lucy’s remark, ‘…I don’t know because what if it was born prematurely?…’ was an example of participants’ awareness of needing to draw a distinction between premature newborns and artificially gestated entities. Whilst Lucy did settle on making a developmental contrast between an entity being in a fully ex-utero environment and an in-utero-like environment, others suggested that a new definition would be needed to solidify the distinction:

I think you would have to re-categorise it because then, like we were saying before, a foetus at the moment has no rights to life. So, that women can have abortions. Women aren’t prosecuted if they do things like smoke, take drugs or alcohol during pregnancy until that child is born at no matter what gestation. But then you’ve got this singularity of a foetus/child that’s not inside its mum but is still classed and cared for as if it was foetus. So, I think you would have to reclassify it. (sic) (Francesca, Midwife)

Francesca’s stance suggested some reliance on the environmental location in her expression of the artificially gestated entity being cared for ‘as if it was a foetus’. However, the earlier part of Francesca’s comment also gave some authority to the bodily-location approach in recognizing the ‘singularity’ of the entity now that is not within the pregnant person’s body. The significance of this singularity, for Francesca, was that this delineates what the (formerly) pregnant person could or could not do to the entity and whether it holds rights. English law is clear that legal personhood begins following live birth, and this is considered as a means to provide protection to pregnant people and their bodily integrity.^68^ Despite controversy over the moral status of the foetus or the arbitrary nature of birth as a significant milestone,^69^ it is conceded that, for legal purposes, it is a practical defining line and is important for legal regulation.^70^ Francesca’s position therefore upholds this legal relevance and seeks to not necessarily select one of the location approaches to define the entity, but rather to incorporate them both within a new categorization.

Although Francesca’s approach provides more nuance in defining the artificially gestated entity, it still begs the question as to why an artificially gestated entity cannot be treated as a newborn if there is a desire to give it the protections afforded to those born alive. Devin’s reflection illuminated this:

I suppose there would have to have some definite legal distinction about what is termed a newborn and what an in-utero baby is. Because at the moment in the UK a baby doesn’t count as a separate individual from the mother until the head is delivered. So, if the baby was still technically in utero I suppose it would have the same rights as an unborn baby in a human, which is nothing really. (Devin, Midwife)

Devin refers to the requirement of birth for the entity to be considered a separate individual and yet acknowledges that entities ‘technically’ within an in-utero environment would not meet this requirement and therefore would have no rights. There is also some confusion as to what Devin meant by ‘in-utero baby’. First, Devin stated that a legal distinction between a newborn and an in-utero baby is needed, which suggests that an in-utero baby is not a foetus, as this distinction between the two already exists. However, Devin then went on to state that an in-utero baby would have the same (non) rights as an ‘unborn baby’, otherwise known as a foetus. Like Francesca, it appeared that Devin wanted to use a new legal distinction to differentiate the artificially gestated entity from a foetus in order for it to have some protections, yet was still giving weight to its in-utero-like environment to distinguish it from a newborn.

The participants’ desire for a new categorization or distinction highlighted that the current legal context cannot accommodate a combination of the bodily and environmental location. The current legal framework in England is not equipped to regulate an entity outside of the human body ‘cared for as if it is a foetus’. For some participants, it was unsatisfactory to simply consider the entity as a foetus, for it would not be considered a legal person with rights or protections, and similarly, defining it as a newborn would not accurately correlate with its in-utero environment. In seeking to manage this pull between designating the entity as a foetus or a newborn, Leia suggested the need for an “interim definition”:

I don’t think that we know enough about it to be able to accurately say whether it is still a foetus or a newborn, but my gut feeling is that we would probably need an interim definition or something between foetus and newborn, something new that defines exactly what it is or describes exactly what it is. (Leia, Consultant Neonatologist)

Despite Leia not dismissing the possibility that the artificially gestated entity could be a foetus or a newborn, they acknowledged that a middle ground may be found in aligning the entity between the two options. The use of an interim definition, as suggested by Leia, may act as an appropriate bridge for Kingma and Finn’s distinction between the characteristics the artificially gestated entity shares with both a foetus and a newborn,^71^ and reflects the position of Dutch healthcare professionals who similarly considered a ‘transitional status’ for the entity.^72^ An interim or transitional status could therefore address the likeness that the concept of bodily location draws between the entity and a newborn, as well as acknowledge the resemblance between the entity and a foetus due to their environmental locations.

Attempts have been made to address the issue of terminology when discussing an artificially gestated entity, such as Romanis’s term ‘gestateling’ used to refer to ‘a developing human being in the process of ex utero gestation’.^73^ However, Romanis acknowledges that such a term may not provide the conceptual clarity needed in a clinical setting.^74^ Developers of the ‘Biobag’ have used the term ‘fetonate’ or ‘fetal neonate’ to capture the idea that an artificially gestated entity is a ‘neonate kept in a fetal physiological state’.^75^ However, it is questionable whether such terminology is useful to lay people,^76^ and most significantly, such terminology offers little by way of signalling how the entity should be treated in law, particularly since current legal definitions treat a foetus and a newborn in completely different ways.^77^ These attempts therefore do little to satisfy the participants’ desire to redefine the entity in order to secure it some protections. Whilst new terminology may more accurately outline what the entity is, a new term or definition alone may not tell us how the entity is to be treated in both law and medicine.

### D. Practical Implications

The extent to which a definition needs to do much more than simply name the artificial gestated entity became very apparent when participants referred to the practical implications associated with new definitions. Innis pointed out:

…on a really very practical level, if this baby is receiving this treatment it needs an NHS number, and it needs a hospital number. From a really practical point of view, that is an individual because it is not being treated as the mother anymore, or through the mother. So, you know, from a practical point of view, it has to be considered an individual. (Innis, Midwife)

Innis’s statement here indicates the continued relevance of bodily location and the role it plays in medical practice. Rather than using bodily location to allocate a legal status to the entity, in the medical context, it would also give the entity a status as a patient, which encompasses numerous practicalities such as allocating an NHS number. Even if the environmental location is applied here and the entity is considered to still be in an in-utero-like environment and akin to a foetus, a foetal patient entirely separated from the pregnant person would now exist.^78^ Innis, however, proposed that a new definition would be needed, and suggested that emphasis on bodily location could still be relevant without it resulting in the entity being considered born:

Yes, I think there would have to be a new definition that this is a being and a person in their own right, with their own rights. So they are not considered as one with the mother, like they are in utero. But, that it is not born. It is not a date of birth. It is not, from that moment, that it has been born. But I think it would have to be a whole new kind of category, where they have the same rights as an individual… (Innis, Midwife)

Innis, like other participants, was keen to use the entity’s separation from the pregnant person as a reason to secure rights for the artificially gestated entity. However, Innis’s approach to the bodily location was to disconnect its links to birth. Whilst previously the emphasis on bodily location appeared to be driven by the legal significance attached to birth, Innis appeared to suggest that exit from the pregnant person’s body need not be synonymous with birth, but could still be synonymous with the acquisition of rights. Such an approach would require a substantive reconsideration of the legal meaning attached to birth, and Colgrove has suggested that a decision to alter the existing definition of birth must have a compelling reason, ‘given widespread agreement on the term’.^79^

This widespread agreement is, however, brought into question with the prospect of artificial placentas. Even participants who did not seek to actively uncouple the bodily-location approach from birth, as Innis suggested, questioned how birth would be defined with the use of an artificial placenta:

Then I suppose another question…would be what you would do with the gestations. Was that baby born at 23 weeks gestation? Or is the baby continued in its gestation and then when it’s removed from the artificial womb then it’s then reborn in that new gestation so it’s sort of a middle category. I suppose it’s the legal side of it that would define that. (Alma, Midwife)

Similarly, in response to the question as to whether an artificially gestated entity would be considered born, Megan stated:

I think so, yes, but I think there would have to become a new phrase. There needs to be a new way to explain that birth and then to explain when the baby comes out of the womb—the artificial womb. Again, there needs to be a new phrase and understanding for that. (Megan, Midwife)

Marking the point at which the entity is born was brought into question by these participants as they grappled with what it would mean when the entity both entered and exited the artificial placenta. Megan, in particular, was seeking a way to explain this, perhaps having in mind the explanation they would have to give to a pregnant patient. The practicalities of this were of further concern to Tate who pondered:

…whether we would then say in documentation born at say 23 weeks via c-section and then there’s sort of entering into another sphere then… (Tate, Midwife)

With a requirement to record when births take place, participants were thinking about how records would reflect an entity’s gestation in an artificial placenta. These practical concerns illustrate the importance of not only how the entity is defined but also the processes around its gestation. Alma’s comment that ‘it’s the legal side of it that would define that’ suggests that legal definitions will direct their practice.

The need for legal direction was particularly poignant when some participants were quite clear that artificial gestation would change the process of birth:

Researcher: Do you think it [the artificially gestated entity] would be considered almost born again when it came out of the artificial womb?

Wyatt: Definitely, because there’s another transition they’ve got to make, haven’t they? They’re born into a bag and not using their lungs and now they’ve got to use their lungs. It’s like a, instead of a two-stage process, it’s a three-stage process, isn’t it?” (Wyatt, Midwife)

Wyatt’s suggestion of birth becoming a three-stage process reflects participants’ earlier considerations of developmental milestones and the physiological difference between an artificially gestated entity and a newborn. However, rather than designating the final exit from the artificial placenta as the point of birth, Wyatt outlined that two births would take place during the process. Legal clarity would be required here to ascertain the significance, if any, of those births, particularly which would result in the entity’s acquisition of rights. Legal guidance would also be necessary to ascertain how the entities should be treated in medical settings, both in terms of receiving treatment and also in regard to record keeping.

In contrast to Wyatt and other participants, however, for those who considered the artificial placenta akin to an incubator and the artificially gestated entity no different than a newborn, the entity’s exit from the artificial placenta posed no problems:

Researcher: Would you consider it [the artificially gestated entity] to be reborn when it comes out of the artificial womb?

Harley: No, I think because I just see this technology as instead of an incubator. I just see it as- no, I don’t see it as babies being lined up in a lab being grown, I see it as me caring for that baby in a different way than I currently care for it. Does that make sense?” (Harley, Consultant Neonatologist)

In continuing to rely upon the medical context and the correlation between an artificial placenta and an incubator as two types of treatment, Harley’s response minimized any practical concerns deriving from the technology. Mary responded similarly to a question of whether the entity leaving the artificial placenta was significant:

No, because it’s still a machine that it’s in. Like in the NICU units, they’re in an incubator—even, say, from really early, say a 23-, 24-week baby. They’re tiny. They don’t look like a baby. They’re there still, and incubating and gestating, but in that environment, so they’ve been born already, one way or another. (Mary, Midwife)

Despite reference to the entity still ‘gestating’ and not looking like a baby, Mary was firm in her position that the entity had been born and, like Harley, relied upon considering the artificial placenta as a machine like those that already exist in neonatal intensive care units.

In very stark contrast to Wyatt, who proposed a new three-stage process of birth, Jayne quite vividly situates the use of an artificial placenta into existing medical practices when it comes to birth:

…at the moment, a baby goes into special care, they go into an incubator, all their stuff is put on high, then it’s reduced, reduced, reduced and then they graduate to the next room where it’s less of an incubator, like half incubator and then they graduate to the last room which is a cot, basically a cot and then they spend a bit of time in there and then they go home. So it’s the same principle really, you’d probably just add another stage to it, wouldn’t you? They’d probably go from an artificial womb to an incubator, to a half incubator, to a cot. (Jayne, Midwife)

Although Jayne did add ‘another stage’ to the process, the process to which they were concerned was the treatment provided after the birth had taken place. The technology is simply slotted into an already existing schedule of events that occur around neonatal care. From this perspective, there is minimal disruption to the legal or medical context surrounding birth and therefore no call for new definitions or explanations.

Whilst it appears that it is only those who leaned on the environmental location that were concerned with the practical implications of artificial placentas, it is interesting to illustrate how these implications caused one participant to question their position on the artificial placenta being akin to a ‘more advance incubator’ and the entity being considered to be born:

Well, I would’ve thought because it’s still enclosed in an, I know this is contradicting what I’m saying, but I think because it’s in a sealed unit, whatever way that’s going to be, I think…*sigh*, my first gut reaction is that no it’s not born. This is where, when do you say its birthday is then? Who is going to decide when its birthday is, if you’re not going to term that as when it is born? Yeah, I don’t know. (Freda, Midwife)

Freda’s position on the artificial placenta being like an incubator seemed to waver when they spoke of the ‘sealed’ nature of the device and served as a clear example of a move between reliance on the bodily location to the environmental location.^80^ Despite then determining that the entity would not be considered born, Freda’s position became destabilized again when considering the practicality of recording the birth and designating a birthday. Freda’s wavering is a very clear illustration of the difficulty in balancing the bodily and environmental approaches, but also indicates that practical considerations contribute to determining how the artificially gestated entity is treated.

Even if it is the case that artificial placentas are smoothly implemented as a simple extension of neonatal intensive care, clarity will still be needed as to how the entity, during its period of artificial gestation, should be treated and how medical records should reflect this.

## IV. DISCUSSION

The theme presented in this article has illustrated the stark views expressed by healthcare professionals in relation to the status of the artificially gestated entity. These contrasting views echo that of the disagreement circulating in academic debates.^81^ Wozniak and Fernandes, for example, believe that artificial placenta technology is no different from neonatal intensive care as both seek to mimic the human placenta with noise and light restrictions.^82^ However, Romanis argues against comparisons between artificial placentas and neonatal intensive care on the basis that a newborn placed in neonatal intensive care is exposed and can be interactive with the social environment.^83^ The factors relied upon to support both academic assertions are arguably sound, but lead to very different conclusions. Similarly, some healthcare professionals in this study lean upon legal definitions of birth to defend a reliance on bodily location to consider the artificially gestated entity a neonate, whilst others consider the in-utero-like environment of the artificial placenta to make the entity akin to a foetus. While reliance on both legal definitions and medical understandings may be justified, the fact that these frameworks give rise to such disparate definitions of an artificially gestated entity illustrates how ill-equipped they are to respond to this new technology. This is further evidenced by participants who called for new or interim definitions.

A lack of agreement amongst healthcare professionals in regards to artificial placentas was also evident in Australia in relation to whether and how partial ectogestation should impact abortion practice.^84^ A study in the Netherlands additionally shares strikingly similar findings to those described in this article.^85^ A lack of consensus amongst Dutch healthcare professionals mirrored the pull between those opting to recognize an artificially gestated entity as a neonate due to it ‘being born’ and those who considered the entity to be transferred to a ‘fluid-based environment’ and therefore aligned with a foetus.^86^ The Dutch study included more neonatal nurses and specialists, suggesting that the lack of consensus among healthcare professionals in England is not simply due to the high number of midwives represented in this study. Rather, a lack of consensus is borne from how the technology itself is viewed and described,^87^ which is informed by existing legal and medical frameworks.

Reliance upon current legal understandings of birth and the born-alive rule has already been questioned academically.^88^ For example, the legal arbitrariness of birth has been criticized prior to the advent of artificial placentas,^89^ and the current legal position has arguably only been taken because the exit of a foetus from the pregnant person’s body is the easiest and most identifiable stage, whereby a fully separate individual can be seen.^90^ In this respect, birth has been used as a convenient ‘bright line’ marker to signify the beginning of legal personhood,^91^ which has been reinforced within medical practice despite technological advances now giving us the ability to confirm foetal existence prior to birth.^92^ The ongoing academic debate, now coupled with the results of this study, indicates that the current legal framework is not fit for purpose and cannot adequately provide cohesive responses to the questions artificial placentas pose, such as how to define an artificially gestated entity. This leaves the future regulation of the technology in question and underscores the pressing need for legislative reform before the technology is implemented into clinical practice.

Several suggestions have been made as to alternative constructions of the law. Steiger, by example, has suggested that legal status must be contingent on a developmental stage as opposed to location to avoid inconsistencies, regardless of the gestational method.^93^ Alternatively, Abecassis suggests that there should be a variety of statuses, differing between pre- and post-implantation and intracorporeal and extracorporeal gestation, to allow for the law to respond to complex situations involving embryos and foetuses.^94^ Alghrani similarly suggests that a distinction can be drawn between an in vitro foetus and an in vivo foetus because the former is independent of the body of an autonomous being.^95^ Healthcare professionals in this and other studies^96^ have suggested interim definitions to bridge the legal gap between foetuses and neonates where an artificially gestated entity may fall.

Of note is how some legal definitions, particularly in relation to birth, arguably rely upon medical determinations. Romanis, for example, has suggested that the broad description of ‘any other signs of life’ in determining stillbirth in the Birth and Deaths Registration Act 1953 may be an indication of Parliament’s reliance on medical input.^97^ Advances in medical technologies and the medicalization of pregnancy and childbirth^98^ have also influenced legal thresholds, such as the lowering of the time limit when terminations of pregnancy can be justified under section 1(1)(a) of the Abortion Act 1967. Whilst the healthcare professionals in this study seem to defer to legal guidance as to the status of an artificially gestated entity, it is likely that a legal analysis alone will not be sufficient to resolve the issue and that regulations may incorporate some deference to medical opinion. However, the lack of consensus evident in this study illustrates that artificial placenta technology presents new ground for the medical as well as the legal profession. One therefore cannot simply rely on the other to fill regulatory gaps. Rather, legal regulation needs to account for its implications on clinical practicalities, and clinical practices need to align with the law. For developers of artificial placenta devices who assume that the entity is no different from a premature newborn (albeit kept in a foetal physiological state),^99^ the presumption that current clinical practice will translate neatly to the artificial placenta is brought into question by this study. Whilst some healthcare professionals constructed the artificial placenta device as another form of treatment within neonatal intensive care, this view was not shared throughout the participant pool. The day-to-day implications on clinical practice raised, such as with regard to record keeping, speak to the importance of stakeholder engagement as the technology develops. Beyond settling a legal definition of the artificially gestated entity, careful attention must be paid to the practical consequences for clinical practice, as this will impact how healthcare professionals record, manage, and care for artificially gestated entities.^100^

Interestingly, and unlike the participants in this study, academics have also considered how the way the artificially gestated is framed may depend on how it is received by people. For example, Romanis suggests that the lack of interactivity that the entity has with its surroundings may influence how others feel towards it.^101^ Raskin and Mazor similarly claim that visibility of an ‘ex utero foetus’ will alter its moral status, particularly as it becomes more recognizable as a human.^102^ This could, in turn, impact how it is perceived socially and legally. Kendal, in their exploration of visual bioethics and artificial placentas, similarly acknowledges that increased visuals may enhance bonding between parents and the artificially gestated entity, but could also generate feelings of repulsion and ultimate rejection of the technology.^103^ It is of note that none of the participants drew upon the potential perspectives of patients (such as the formerly pregnant person) to help them determine their definitions of the artificially gestated entity. Arguably, the participants’ focus on the entity itself was a product of the specific question under consideration (despite other parts of the interviews discussing the pregnant person^104^) and artificial placentas being developed as a form of treatment for premature neonates. Nevertheless, it emphasizes the significant role that the legal and medical context played in their deliberations and how this potentially overshadowed consideration of parental perspectives. A study considering the views of other key stakeholders, such as parents with neonatal intensive care experiences,^105^ would help determine whether they similarly defer to legal and medical frameworks when defining the artificially gestated entity, or whether other contexts or frameworks may offer solutions that the legal and medical frameworks are unable to deliver.

The weight given by the participants in this study to current legal and medical contexts should also serve as a warning as to the way in which legal regulation can shape interpretations of new technologies. The desire of participants to fit the concept of an artificially gestated entity into current legal and medical contexts perhaps hindered some of them from embracing new possibilities. Those who suggested a new or interim definition were more open to the possibility of artificial placentas disrupting our current understandings of birth and personhood. Whilst it may be easier to make artificial placentas fit within current contexts, new technologies should also be embraced as an opportunity to challenge and question existing concepts. Whilst understandings of birth are arguably widespread, this may simply be the case because those understandings have never had reason to be challenged. Similar complexities were mirrored when in vitro fertilization (IVF) developed, and it became possible to culture embryos outside of the human body. As with artificial placentas, IVF presented a previously uncharted territory whereby an extensive amount of time can now exist between fertilization of gametes and implantation of embryos. Although neither process invites a status of legal personhood, new legislation has been drafted to govern the use of embryos in clinics that can now exist outside of the body.^106^ Additionally, embryos are an example of an entity that does not have legal personhood but nevertheless receives legal protections.^107^ Therefore in a similar manner, new regulations beyond our current frameworks are needed to govern how an entity, gestating artificially outside of the human body, is to be treated.

The lack of consensus amongst healthcare professionals, particularly if found to be more widespread, indicates that further discussion and consultation as to the status of an artificially gestated entity is needed, rather than relying on assumptions that existing frameworks can work around the technology. Whilst several calls have been made for further empirical work and public consultation on the issue of artificial placentas, these have largely been aimed at ethicists and legal professionals.^108^ However, to ensure that medical practice is consistent, clear, and aligned with legal requirements, healthcare professionals need to remain within this sphere of consultation.^109^

## Conclusion

As artificial placenta technology continues to develop, engagement with key stakeholders becomes increasingly urgent to ensure it meets the needs of its users and gains public support. As intermediaries between patients and developers, healthcare professionals will play a significant role in shaping how the technology is perceived and therefore supported.

A central issue of contention is how to define the entity that comes to be gestated in an artificial placenta. In this study, healthcare professionals were unable to reach a consensus on this point. Their reliance upon legal and medical frameworks, whether favouring a bodily-location approach, an environmental-location approach, or proposing an interim definition, underscored the inadequacy of current models to resolve the issue. In addition, engagement with healthcare professionals illustrated the practical implications of ambiguous definitions of the artificially gestated entity.

As evidenced in this article, artificial placenta technology introduces unchartered territory for both legal and medical professionals, who will need to work together to decide how artificial placentas and the entities that gestate within them will be regulated. However, this study suggests that continued reliance on existing frameworks will do little to yield consensus amongst key stakeholders. This lack of agreement may then hinder broader support for the technology as it develops.

Moving forward, broad stakeholder consultation must continue to develop new frameworks that can appropriately respond to artificial placenta technology and the new legal and medical challenges it poses.

## Footnotes

1 Stacy Beck and others, ‘The Worldwide Incidence of Preterm Birth: A Systematic Review of Maternal Mortality and Morbidity’ (2010) 88 Bulletin of the World Health Organization 31; Megan Coughlin and others, ‘An Artificial Placenta Protects Against Lung Injury and Promotes Continued Lung Development in Extremely Premature Lambs’ (2019) 65 ASAIO Journal 690.

2 Emily A Partridge and others, ‘An Extra-uterine System to Physiologically Support the Extreme Premature Lamb’ (2017) 8 Nature Communications 1; Haruo Usuda and others, ‘Successful Maintenance of Key Physiological Parameters in Preterm Lambs Treated with Ex Vivo Uterine Environment Therapy for a Period of 1 Week’ (2017) 217 American Journal of Obstetrics and Gynecology 457.e1; E Joanne Verweij and others, ‘Ethical Development of Artificial Amniotic Sac and Placenta Technology: A Roadmap’ (2021) 9 Frontiers in Pediatrics 793308.

3 Jennifer McLeod and others, ‘Splenic Development and Injury in Premature Lambs Supported by the Artificial Placenta’ (2019) 54 Journal of Pediatric Surgery 1147.

4 Partridge and others (n 2); Matther Hornick and others, ‘Technical Feasibility of Umbilical Cannulation in Midgestation Lambs Supported by the EXTra‐Uterine Environment for Neonatal Development (EXTEND)’ (2019) 43 Artificial Organs 1154; Felix De Bie and others, ‘Artificial Placenta and Womb Technology: Past, Current, and Future Challenges Towards Clinical Translation’ (2021) 41 Prenatal Diagnosis 145.

5 Children’s Hospital of Philadelphia, ‘A Unique Womb-Like Device Could Reduce Mortality and Disability for Extremely Premature Babies’ (CHOP, April 24 2017) <https://www.chop.edu/news/unique-womb-device-could-reduce-mortality-and-disability-extremely-premature-babies> accessed 25 July 2025.

6 Rachael Robertson, ‘FDA Advisors Grapple with Logistical, Ethical Issues of Artificial Womb Technology’ (*MedPageToday*, 15 September 2023) <https://www.medpagetoday.com/obgyn/pregnancy/106426> accessed 22 July 2025.

7 Max Kozlov, ‘Human Trials of Artificial Wombs Could Start Soon. Here’s What You Need to Know’ (2023) 621 Nature 459.

8 For clarity, reference to an artificially gestated entity throughout the article will mean an entity that has undergone partial human gestation and partial gestation in an artificial placenta. Discussions of complete ectogestation, whereby an embryo is placed within an artificial placenta for the entire duration of gestation, are beyond the scope of this article.

9 Nick Colgrove, ‘Subjects of Ectogenesis: Are ‘Gestatelings’ Fetuses, Newborns or Neither?’ (2019) 45 Journal of Medical Ethics 723.

10 Felix R De Bie and others, ‘Ethics Considerations Regarding Artificial Womb Technology for the Fetonate’ (2022) 23 American Journal of Bioethics 67.

11 Elizabeth C Romanis, ‘Artificial Womb Technology and the Significance of Birth: Why Gestatelings are not Newborns (or Fetuses)’ (2019) 45 Journal of Medical Ethics 727; Elselijn Kingma and Suki Finn, ‘Neonatal Incubator or Artificial Womb? Distinguishing Ectogestation and Ectogenesis Using the Metaphysics of Pregnancy’ (2020) 34 Bioethics 354.

12 De Bie and others (n 10).

13 ibid 68.

14 See eg, IG Cohen, ‘Artificial Wombs and Abortion Rights’ (2017) 47 Hastings Center Report; Joona Räsänen, ‘Ectogenesis, Abortion and a Right to the Death of the Fetus’ (2017) 31 Bioethics 697 and Claire Horn, ‘Ectogenesis is for Feminists: Reclaiming Artificial Wombs from an Anti-abortion Discourse’ (2020) 6 Catalyst: Feminism, Theory, Technoscience 1.

15 Peter Singer and Deanne Wells, ‘Ectogenesis’ in Scott Gelfand and John R. Shook (eds), *Ectogenesis: Artificial Womb Technology and the Future of Human Reproduction* (Rodopi 2006) 11–13.

16 Horn (n 14) 4.

17 Jonathan Herring, *Law Through the Life Course* (Policy Press 2021) 57. Evidence of this can be seen with the 14-day limit placed on embryo research to take account of public concerns whilst also not impeding scientific progress (Natasha Hammond-Browning, ‘Ethics, Embryos, and Evidence: A look Back at Warnock’ (2015) 23 Medical Law Review 588, 605).

18 Eliot Freidson, *Profession of Medicine: A Study of the Sociology of Applied Knowledge* (The University of Chicago Press 1988) 248–250; Talcott Parsons, *The Social System* (Routledge 2013) ch 10.

19 Arianne Shahvisi, ‘Conscientious Objection: A Morally Insupportable Misuse of Authority’ (2018) 13 Clinical Ethics 82, 83.

20 Tess Johnson and Elizabeth C Romanis, ‘The Relationship Between Speculation and Translation in Bioethics: Methods and Methodologies’ (2023) 41 Monash Bioethics Review 59.

21 Elizabeth C Romanis, ‘Equality‐enhancing Potential of Novel Forms of Assisted Gestation: Perspectives of Reproductive Rights Advocates’ (2023) 37 Bioethics 637 and Elizabeth C Romanis, ‘The Law is Very, Very Outdated and Not Keeping Up with the Technology’: Novel Forms of Assisted Gestation, Legal Challenges, and Perspectives of Reproductive Rights Advocates in England and Wales (2023) 10 Journal of Law and the Biosciences lsad027.

22 Details of a study funded by the Future of Human Reproduction Interdisciplinary team can be found at <https://www.lancaster.ac.uk/future-of-human-reproduction/funding-schemes/> accessed 25 July 2025.

23 Lydia Di Stefano and others, ‘Ectogestation Ethics: The Implications of Artificially Extending Gestation for Viability, Newborn Resuscitation and Abortion’ (2020) 34 Bioethics 371.

24 Angret de Boer and others, ‘Human Artificial Placenta Technology-Trials: Counselling and Informed Consent Using Healthcare Professionals’ and Parental Perspectives’ [2025] Pediatric Research 1 and Angret de Boer and others. ‘Stakeholder Perspectives on the Design of First‐In‐Human Trials for Artificial Amnion and Placenta Technology: A Qualitative Study’ [2025] BJOG: An International Journal of Obstetrics & Gynaecology 1.

25 Elizabeth C Romanis, ‘Challenging the ‘Born Alive’ Threshold: Fetal Surgery, Artificial Wombs, and the English Approach to Legal Personhood’ (2020) 28 Medical Law Review 93, 112.

26 Partridge and others (n 2).

27 Nigel Fielding and Hilary Thomas, ‘Qualitative Interviewing’ in Nigel Gilbert and Paul Stoneman (eds), *Researching Social Life* (4th edn, SAGE 2016) 283–284.

28 Virginia Braun and Victoria Clarke, ‘Using Thematic Analysis in Psychology’ (2006) 3 Qualitative Research in Psychology 77; Virginia Braun and Victoria Clarke, *Successful Qualitative Research: A Practical Guide for Beginners* (SAGE 2013); Virginia Braun and Victoria Clarke, *Thematic Analysis: A Practical Guide* (SAGE 2022).

29 Braun and Clarke, ‘Using Thematic Analysis’ ibid 79.

30 Virginia Braun and Victoria Clarke, ‘Reflecting on Reflexive Thematic Analysis’ (2019) 11 Qualitative Research in Sport, Exercise and Health 589, 592; Braun and Clarke, *Thematic Analysis* (n 28).

31 Braun and Clarke, *Thematic Analysis* (n 28) 12–22.

32 ibid 12–22.

33 Virginia Braun and Victoria Clarke, ‘Can I Use TA? Should I Use TA? Should I Not Use TA? Comparing Reflexive Thematic Analysis and Other Pattern‐based Qualitative Analytic Approaches’ (2021) 21 Counselling and Psychotherapy Research 37, 39.

34 Braun and Clarke, ‘Using Thematic Analysis in Psychology’ (n 28) 83.

35 ibid 84–85.

36 Braun and Clarke, *Successful Qualitative Research* (n 28) 223–224.

37 Braun and Clarke, *Thematic Analysis* (n 28) 85–87.

38 Note that the terminology most prominently in circulation at the time of the study was ‘artificial womb’ and the process it undertakes ‘partial ectogenesis’ and these were the terms used during the interviews and may appear in quotes from participants. For clarity an ‘artificial womb’ in this study is referring to the same device now more appropriately referred to as an artificial placenta or artificial amnion and placenta technology and references to partial ectogenesis correlates with what is now termed partial ectogestation (See Kingma and Finn (n 11) and EJ Verweij and Elselijn Kingma, ‘Artificial Placenta–Imminent Ethical Considerations for Research Trials and Clinical Translation’ (2023) 23 American Journal of Bioethics 85).

39 Births and Deaths Registration Act 1953, s 2.

40 s 1.

41 s 41.

42
*Paton v British Pregnancy Advisory Service Trustees* [1979] QB 276, 279.

43
*Attorney-General’s Reference (No 3 of 1994)* [1998] AC 245.

44 Births and Deaths Registration Act 1953, s 41 as amended by Still-Birth (Definition) Act 1992 s 1(1).

45 Births and Deaths Registration Act 1953, s 1 and s 41 as amended by Still-Birth (Definition) Act 1992 s 1(1).

46 For example, they cannot bring an action in tort or have someone do so on their behalf- *Burton v Islington Health Authority* [1993] QB 204.

47 Although parents can now request a baby loss certificate, this is not considered a legal document-NHS, ‘Request a baby loss certificate’ (*NHS*) <https://www.gov.uk/request-baby-loss-certificate> accessed 22 July 2025.

48 It is acknowledged that a further discussion as to how the death of an artificially gestated entity would be defined may have led participants to reconsider their position (Anna Nelson and others, ‘Death and the Artificial Placenta’ (2023) 11.2 Journal of Law and the Biosciences lsae013); however, such considerations were beyond the scope of this study.

49 Mary Anne Warren, *Moral Status: Obligations to Persons and Other Living Things* (Clarendon Press 1997) 217.

50 Even though the pregnant person and the foetus remain connected for a short while after birth, this continued umbilical connection following expulsion from the pregnant person’s body has not historically impacted whether that foetus is considered born in English law (Romanis, ‘Challenging the “born alive” threshold’ (n 25) 105).

51 See Elizabeth C Romanis, ‘Artificial Womb Technology and the Frontiers of Human Reproduction: Conceptual Differences and Potential Implications’ (2018) 44 Journal of Medical Ethics 751 and Kingma and Finn (n 11).

52 Singer and Wells (n 15) 9–10.

53 Haruo Usuda and others, ‘Artificial Placenta Technology: History, Potential and Perception’ (2023) 141 Placenta 10, 15.

54 When transferring a foetus to an artificial placenta, it will be given a paralytic to prevent the lungs from emptying of fluid, and oxygenation and gas exchange will occur in the artificial placenta via blood circulation

55 Amel Alghrani and Margaret Brazier, ‘What Is It? Whose It? Re-Positioning the Fetus in the Context of Research?’ (2011) 70 Cambridge Law Journal 51, 72.

56 Colgrove provided this as: ‘…the complete expulsion or extraction from its mother of a product of conception, irrespective of the duration of the pregnancy, which, after such separation, breathes or shows any other evidence of life—for example, beating of the heart, pulsation of the umbilical cord or definite movement of voluntary muscles—whether or not the umbilical cord has been cut or the placenta is attached’ (Colgrove (n 9) 724).

57 ibid 2.

58 Usuda and others (n 53) 15.

59 Romanis (n 51) 753–754. Romanis (n 11) 728.

60 British Association of Perinatal Medicine, *Perinatal Management of Extreme Preterm Birth before 27 Weeks of Gestation: A Framework for Practice* (London, October 2019) <https://hubble-live-assets.s3.amazonaws.com/bapm/attachment/file/182/Extreme_Preterm_28-11-19_FINAL.pdf> accessed 26 October 2022.

61 Kingma and Finn (n 11).

62 ibid 358.

63 The amnion is a membrane covering the embryo, which fills with amniotic fluid as the embryo develops into a foetus. The chorion is the outer membrane that contributes to the development of the placenta.

64 They additionally use their conceptualisation to distinguish between artificial womb technology and neonatal intensive care, arguing that the former focuses on the physiological blueprint of a foetus and the latter on the physiological blueprint of a newborn.

65
*Paton v British Pregnancy Advisory Service Trustees* [1979] QB 276, 279.

66 S 1(1).

67 I am grateful to the anonymous reviewer who raised this point.

68 Romanis (n 25) 99–100. US case law indicates how moving away from the born-alive rule has resulted in pregnant people being found guilty of crimes against the unborn (Agota Peterfy, ‘Fetal Viability as a Threshold to Personhood: A Legal Analysis’ (1995) 16 Journal of Legal Medicine 607, 627).

69 Warren (n 39) 216.

70 Peter Singer, *Practical Ethics* (3rd edn, CUP 2011) 130; Raanan Gillon, ‘Is There a “New Ethics of Abortion”?’ (2001) 27 Journal of Medical Ethics ii5, ii8.

71 Kingma and Finn (n 11).

72 de Boer and others, ‘Human artificial Placenta Technology-Trials’ (n 24) 2.

73 Romanis (n 51) 751.

74 Elizabeth C Romanis, ‘The Ethical and Legal Status of ‘Fetonates’ Or ‘Gestatelings’’ (2023) 23 American Journal of Bioethics 90, 91.

75 De Bie and others (n 10) 2.

76 Romanis (n 74) 90–91 and Seppe Segers and Elizabeth C Romanis, ‘Ethical, Translational, and Legal Issues Surrounding the Novel Adoption of Ectogestative Technologies’ (2022) 15 Risk management and healthcare policy 2207, 2213.

77 ibid 91.

78 This may be distinguishable from a ‘foetal operate’ who is undergoing surgery with the intention of being returned to the pregnant person’s uterus (Romanis (n 25) 99–100).

79 Colgrove (n 9) 724.

80 It is possible that Freda’s position and other participants who relied on the environmental location may have felt differently if a non-sealed version of an artificial placenta were under discussion, however, the sealed ‘Biobag’ was the most popular system under discussion at the time of the study and seems closest to clinical translation.

81 For eg, see Romanis (n 51); Kingma and Finn (n 11); Colgrove (n 9); Romanis (n 11); Nick Colgrove, ‘Artificial Wombs, Birth and ‘Birth’: A Response to Romanis’ (2020) 46 Journal of Medical Ethics 554.

82 Phillip S Wozniak and Ashley K Fernandes, ‘Conventional Revolution: The Ethical Implications of the Natural Progress of Neonatal Intensive Care to Artificial Wombs’ (2021) 47 Journal of Medical Ethics e-54.

83 Romanis (n 51) 754.

84 Di Stefano and others (n 23) 379.

85 Angret de Boer and others, ‘Human Artificial Placenta Technology-Trials’ (n 24).

86 ibid 2–3.

87 ibid 3–4.

88 Romanis (n 25).

89 Elizabeth Day, ‘Infanticide is Justifiable in Some Cases, Says Ethics Professor’ *The Telegraph* (London, 25 January 2004) <https://www.telegraph.co.uk/news/uknews/1452504/Infanticide-is-justifiable-in-some-cases-says-ethics-professor.html> accessed 9 April 2019.

90 Jonathan Glover, *Causing Death and Saving Lives: The Moral Problems of Abortion, Infanticide, Suicide, Euthanasia, Capital Punishment, War and Other Life-or-Death Choices* (Penguin UK 1990), 126; Romanis (n 25) 100.

91 Herring (n 17) 57. Although the bright line approach has been defended on both medical and moral grounds- see Achas K Burin, ‘Beyond Pragmatism: Defending the ‘Bright Line’ of Birth’ (2014) 22 Medical Law Review 494.

92 Peterfy (n 68) 627.

93 Eric Steiger, ‘Not of Woman Born: How Ectogenesis Will Change the Way We View Viability, Birth, and the Status of the Unborn’ (2010) 23 Journal of Law & Health 143, 158.

94 Marion Abecassis, ‘Artificial Wombs: The Third Era of Human Reproduction and the Likely Impact on French and US Law’ (2016) 27 Hastings Women’s Law Journal 3, 16.

95 Amel Alghrani, *Regulating Assisted Reproductive Technologies: New Horizons* (CUP 2018) 172.

96 Angret de Boer and others, ‘Human Artificial Placenta Technology-Trials’ (n 24).

97 Romanis (n 25) 112.

98 Denis P Gray, Eleanor White and Ginny Russell, ‘Medicalisation in the UK: Changing Dynamics, But Still Ongoing’ (2016) 109 Journal of the Royal Society of Medicine 7, 7; Karen Ballard and Mary A Elston, ‘Medicalisation: A Multi-Dimensional Concept’ (2005) 3 Social Theory & Health 228, 232; Peter Conrad, ‘Medicalization and Social Control’ (1992) 18 Annual Review of Sociology 209, 216; Sally Sheldon, *Beyond Control: Medical Power and abortion Law* (Pluto Press 1997).

99 See Di Bie and others (n 10) 68 and Alan W Flake and others, ‘The Artificial Placenta and EXTEND Technologies: One of These Things is Not Like the Other’ (2023) 43 Journal of Perinatology 1343.

100 The issue of liability for healthcare professionals is another issue, which has been raised academically: Romanis (n 25) 118; Victoria Adkins, ‘The Impact of Ectogenesis on the Medicalisation of Pregnancy and Childbirth’ (2021) 47 Journal of Medical Ethics 239, 242; Segers and Romanis (n 76) 2214.

101 ibid.

102 Joyce M Raskin and Nadav A Mazor, ‘The Artificial Womb and Human Subject Research’ in Scott Gelfand and John R Shook (eds), *Ectogenesis: Artificial Womb Technology and the Future of Reproduction* (Rodopi 2006) 167–168.

103 Evie S Kendal, ‘Form, Function, Perception, and Reception: Visual Bioethics and the Artificial Womb’ (2022) 95 The Yale Journal of Biology and Medicine 371, 375.

104 Forthcoming publications of the other themes generated for the study will speak to the consistent foetal focus of the participants throughout the interviews.

105 See n 22.

106 Human Fertilisation and Embryology Act 1990 (as amended by the Human and Fertilisation and Embryology Act 2008).

107 Romanis (n 25) 120.

108 Scott Gelfand, ‘Introduction’ in Scott Gelfand and John R Shook (eds), *Ectogenesis: Artificial Womb Technology and the Future of Reproduction* (Rodopi 2006) 3; Jessica H Schultz, ‘Development of Ectogenesis: How Will Artificial Wombs Affect the Legal Status of a Fetus or Embryo’ (2009) 84 Chicago-Kent Law Review 877, 883; André Krom and others, ‘Capabilities and Stakeholders–Two Ways of Enriching the Ethical Debate on Artificial Womb Technology’ (2023) 23 American Journal of Bioethics 110, 112.

109 Adkins (n 100) and Victoria Adkins, ‘The Warnock Report and Partial Ectogestation: Retracing the Past to Step into the Future’ (2023) 31 Medical Law Review 424.

